# Mathematical modeling of gonadotropin-releasing hormone signaling

**DOI:** 10.1016/j.mce.2016.08.022

**Published:** 2017-07-05

**Authors:** Amitesh Pratap, Kathryn L. Garner, Margaritis Voliotis, Krasimira Tsaneva-Atanasova, Craig A. McArdle

**Affiliations:** a Department of Mathematics, College of Engineering, Mathematics and Physical Sciences, University of Exeter, Exeter, EX4 4QF, UK; b EPSRC Centre for Predictive Modeling in Healthcare, University of Exeter, Exeter, EX4 4QF, UK; c Laboratories for Integrative Neuroscience and Endocrinology, School of Clinical Sciences, University of Bristol, Whitson Street, Bristol, BS1 3NY, UK

**Keywords:** GnRH, GPCR, NFAT, ERK, Mathematical modeling, Mutual information

## Abstract

Gonadotropin-releasing hormone (GnRH) acts via G-protein coupled receptors on pituitary gonadotropes to control reproduction. These are G_q_-coupled receptors that mediate acute effects of GnRH on the exocytotic secretion of luteinizing hormone (LH) and follicle-stimulating hormone (FSH), as well as the chronic regulation of their synthesis. GnRH is secreted in short pulses and GnRH effects on its target cells are dependent upon the dynamics of these pulses. Here we overview GnRH receptors and their signaling network, placing emphasis on pulsatile signaling, and how mechanistic mathematical models and an information theoretic approach have helped further this field.

## Highlights

• We describe a mechanistic model for GnRH action via its Gq/11-coupled receptors.
• GnRH is secreted is pulses which can increase signaling efficiency and robustness.
• Maximal responses occur at sub-maximal GnRH pulse frequency.
• This may reflect incoherent feedforward loops but not upstream negative feedback.
• Single cell measures reveal noisy signaling systems with marked information loss.

## 1 GnRH signaling: an overview

GnRH is a hypothalamic decapeptide that mediates central control of reproduction. It acts via receptors (GnRHR) on pituitary gonadotropes to control synthesis and secretion of the two gonadotropin hormones (LH and FSH) that in turn regulate gametogenesis and steroidogenesis in the gonads. LH and FSH are heterodimeric proteins with distinct β-subunits (LHβ and FSHβ) and a common α-gonadotropin subunit (αGSU) that are packaged into vesicles for release from gonadotropes. Acutely, GnRH regulates the exocytotic fusion of these vesicles with the plasma membrane whereas chronically it increases synthesis of gonadotropins and thereby controls vesicle content. There are three distinct forms of the hormone termed GnRH-I (often known simply as GnRH and also known as LHRH), GnRH-II and GnRH-III. The cloned GnRHR, which are members of the rhodopsin-like GPCR family, have been classified into three groups based on sequence homology. All of the cloned mammalian GnRHR are in groups I or II, and the type I GnRHR of humans, rats, mice, pigs, sheep, and horses share >80% amino acid sequence homology (Millar et al., 2004, Morgan and Millar, 2004). Some primates express type II GnRHR (as well as type I GnRHR), but in humans functional type II GnRHR are not expressed (Morgan and Millar, 2004, Stewart et al., 2009). The central control of reproduction is therefore mediated by GnRH-I acting via type I GnRHR, both of which are absolutely essential for mammalian reproduction (Cattanach et al., 1977, Mason et al., 1986, de Roux et al., 1997).

**Fig. 1:**
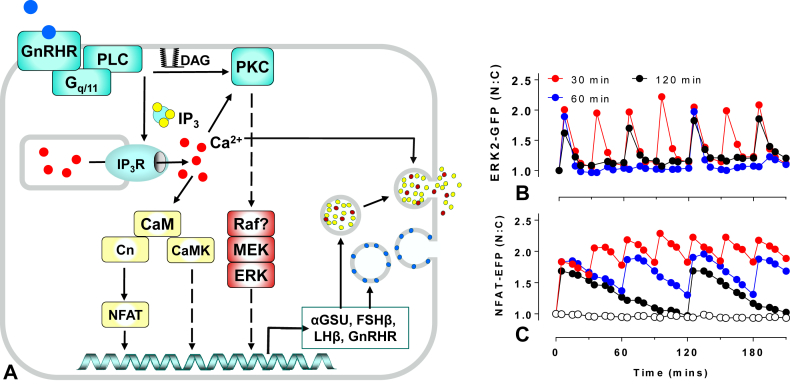
**A simplified GnRHR signaling network**. Panel A: GnRH activates GnRHR causing a Gq/11-mediated activation of phospholipase C (PLC). This generates IP_3_ which drives IP_3_ receptor (IP_3_R)-mediated mobilization of Ca^2+^ from intracellular stores, and diacylglycerol (DAG) which (with Ca^2+^) activates conventional PKC isozymes. GnRH increases cytoplasmic Ca^2+^ and this drives the regulated exocytotic secretion of LH and FSH from within secretory vesicles. Ca^2+^ also activates calmodulin (CaM), which activates CaM-dependent protein kinases (CaMK) and the phosphatase calcineurin (Cn), which activates the Ca^2+^-dependent transcription factor NFAT (nuclear factor of activated T-cells). GnRH also activates MAPK cascades, including the (largely PKC-mediated) activation of the Raf/MEK/ERK cascade shown. NFAT and ERK-activated transcription factors (amongst others) then act in combination to control gene expression. GnRH target genes include the gonadotropin subunits; GnRH acutely regulates the rate of vesicle fusion with the plasma membrane, and chronically regulates the gonadotropin content of these vesicles. Panels B and C: data from HeLa cells transduced to express GnRHR and also ERK2-GFP (B) or NFAT-EFP (C) that translocate from the cytoplasm to the nucleus on activation, providing live cell readouts for the Raf/MEK/ERK and CaM/Cn/NFAT activation, respectively. The data shown are the nuclear:cytoplasmic ratios (N:C) and are from an experiment in which cells received 5 min pulses of 10^−7^ M GnRH at 30, 60 or 120 min intervals. Note that each GnRH pulse causes nuclear translocation of each reporter and the ERK2-GFP translocation responses have more rapid on-set and off-set than the NFAT-EFP responses. Note also that with the highest pulse frequency there is insufficient time for the NFAT-EFP to return to the pre-stimulation value. Similar experiments (and experimental details) are published elsewhere (Armstrong et al., 2009a, Armstrong et al., 2009b, Armstrong et al., 2010).

In gonadotropes, GnRH influences the expression of many genes(Yuen et al., 2002, Yuen et al., 2009, Ruf et al., 2006), although most work in this area focuses on transcription of the gonadotrope signature genes for αGSU, LHβ, FSHβ and GnRHR, all of which are increased by GnRH (McArdle and Roberson, 2015). GnRHR signal primarily via G_q_, which activates PLC to generate IP_3_ and DAG by cleavage of phosphatidylinositol (4,5)-bisphosphate (Fig. 1A). IP_3_ mobilizes Ca^2+^ from intracellular stores and this is followed by Ca^2+^ influx via L-type voltage-gated Ca^2+^ channels. Ca^2+^ then drives the regulated exocytotic secretion of LH and FSH, an effect that is modulated by the concomitant activation of PKC isozymes (Hansen et al., 1987, Hille et al., 1994, Stojilkovic et al., 1991, Zhu et al., 2002). Like many other GPCRs, GnRHR mediate activation of MAPKs including ERK. Mechanisms of ERK activation by GnRH differ between model systems but it is largely mediated by PKC in αT3-1 and LβT2 gonadotrope cell lines (Naor, 2009, Caunt et al., 2006). In rat pituitaries, αT3-1 and LβT2 cells, GnRH also activates JNK (Naor, 2009, Burger et al., 2004, Burger et al., 2009) and p38 (Roberson et al., 1999, Coss et al., 2007) and in LβT2 cells it has been shown to activate ERK5 (Lim et al., 2009). PKC and each of these MAPKs are implicated in control of gonadotropin signature gene expression as described elsewhere (McArdle and Roberson, 2015, Ciccone and Kaiser, 2009, Haisenleder et al., 1991). Several Ca^2+^-regulated proteins are known to mediate transcriptional effects of GnRH. These include calmodulin (CaM), calmodulin-dependent protein kinases, the calmodulin dependent phosphatase calcineurin (Cn) and the Ca^2+^ dependent transcription factor NFAT (McArdle and Roberson, 2015).

## 2 GnRH: a dynamic peptide

GnRH is secreted in pulses that drive pulses of gonadotropin release and are essential for normal reproduction (Dierschke et al., 1970, Clarke and Cummins, 1982). Its effects are dependent on pulse frequency, as shown in early studies where constant GnRH suppressed LH and FSH secretion, whereas restoration of GnRH pulses restored gonadotropin secretion (Belchetz et al., 1978). In humans and other primates, GnRH pulses have a duration of a few minutes and intervals of 30 min to several hours, with pulse frequency differing under different physiological conditions. For example, changes in GnRH pulse frequency drive changes in reproductive status during development, with an increase in pulse frequency driving the increased gametogenesis and gonadal steroid production at puberty (Sisk and Foster, 2004). Similarly, GnRH pulse frequency varies through the menstrual cycle, increasing before ovulation and contributing to generation of the pre-ovulatory gonadotropin surge (Ferris and Shupnik, 2006, Marshall et al., 1993). Moreover, stimulation paradigm is crucial for therapeutic intervention because agonist pulses can maintain or increase circulating gonadotropin levels whereas sustained agonist stimulation (after initial activation) reduces them, causing the chemical castration that is exploited in treatment of breast cancer, prostate cancer and other sex steroid hormone-dependent conditions (Ferris and Shupnik, 2006, Marshall et al., 1993, Bliss et al., 2010). The key observation here is that maximal GnRH effects on gonadotropin secretion are seen at sub-maximal GnRH pulse frequency and this also holds true for effects of GnRH on many of its gene targets, including the signature genes GnRHR, FSHβ and LHβ. Thus physiological and pharmacological control of the system relies on the fact that gonadotropin synthesis and secretion are low when GnRH pulse intervals are too low (i.e. before puberty) or too high (treating constant agonist stimulation as the maximal possible pulse frequency).

## 3 GnRHR: a short tail

It has long been known that sustained agonist exposure causes activation followed by desensitization of GnRH-stimulated gonadotropin secretion, that is not seen with pulsatile stimulation (Belchetz et al., 1978). GnRH causes GnRHR internalization and this could certainly contribute to desensitization of GnRH-stimulated gonadotropin secretion. Sustained stimulation of GPCRs typically causes rapid homologous receptor desensitization, where G-protein receptor kinases phosphorylate Ser and Thr residues, most often within the receptor's COOH-terminal tail, facilitating binding of non-visual arrestins (arrestins 2 and 3). The arrestins prevent G protein activation and target desensitized receptors for internalization, most often via clathrin-coated vesicles (CCVs) (Pierce and Lefkowitz, 2001). Although GnRH was known to induce GnRHR internalization via CCVs (Hazum et al., 1980, Jennes et al., 1984), the cloning of mammalian type I GnRHR revealed most remarkably that it has no COOH-terminal tail (Millar et al., 2004, Tsutsumi et al., 1992, Sealfon et al., 1997). Equally remarkable is the fact that all non-mammalian GnRHR cloned to date have such tails, indicating a period of rapid molecular evolution with the advent of mammals being associated with the loss of COOH-terminal tails. Importantly, it is now established that type I mammalian GnRHR (where explored) do not rapidly desensitize or undergo agonist-induced phosphorylation or arrestin binding. Moreover, although they do show agonist-induced internalization the process is relatively slow and is arrestin-independent (Davidson et al., 1994, Finch et al., 2009, Heding et al., 1998, Hislop et al., 2000, Hislop et al., 2001, McArdle et al., 1999, Vrecl et al., 1998, Pawson et al., 1998). Conversely, non-mammalian GnRHR or type II mammalian GnRHR (with COOH-terminal tails) do undergo agonist induced phosphorylation, arrestin binding and/or arrestin-dependent rapid homologous desensitization and are desensitized and internalized more rapidly than type I mammalian GnRHR. Furthermore, fusing the COOH-terminal of various non-mammalian GnRHR to type I mammalian GnRHR can facilitate rapid desensitization, arrestin binding and internalization (Finch et al., 2009, Hanyaloglu et al., 2001, Heding et al., 1998, Heding et al., 2000, Hislop et al., 2005). The fact that GnRH responses do show homologous desensitization seems initially at odds with the lack of desensitization of type I mammalian GnRHR, but in reality just points to the importance of alternative mechanisms as discussed in more detail below.

## 4 GnRH signaling: a mechanistic modeling approach

**Fig. 2:**
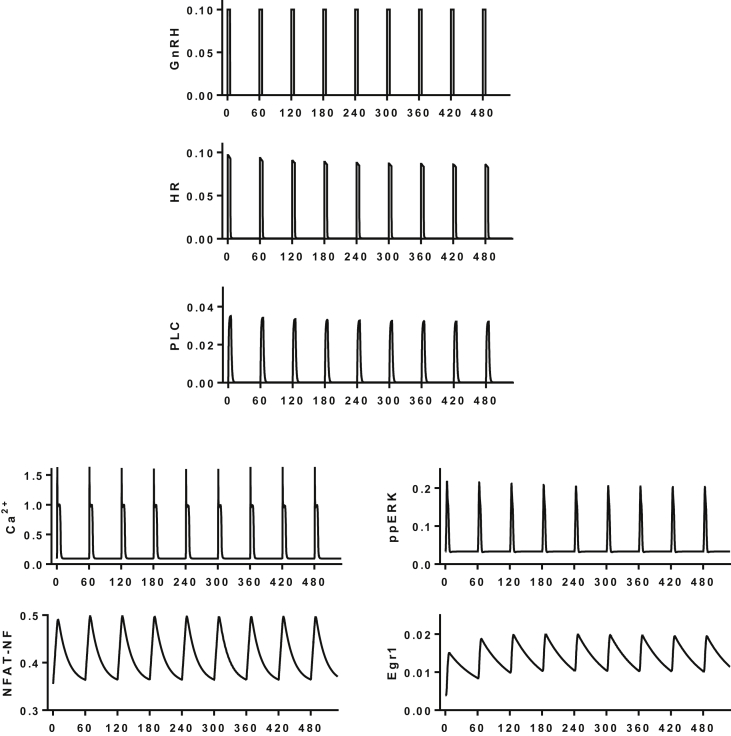
**Simulating GnRH signaling**. The GnRH signaling network has been simulated with a series of thirty-four ordinary differential equations and parameters trained on ERK2-GFP and NFAT-EFP translocation data from HeLa cells transduced with GnRHR (Tsaneva-Atanasova et al., 2012). This model was modified to add agonist-induced GnRHR internalization (and recycling), trained against data from GnRH time-course and concentration-dependence experiments in LβT2 cells (see Supplemental Data) and then used to simulate responses to GnRH pulses. The figure shows system input (square wave pulses of 10^−7^ M GnRH with 5 min width and 60 min period) as well as model-predicted concentrations of hormone-occupied GnRHR (HR), active PLC, cytoplasmic Ca^2+^, nuclear ppERK, nuclear Egr1 (all μM) and the nuclear fraction of NFAT (NFAT-NF). Note that the simulated upstream signals are rapid in onset and offset whereas the downstream responses (NFAT translocation and Egr1 levels) are much slower.

Mathematical modeling of the entire GnRH signaling network would be unrealistic at present, particularly if one were to attempt to overlay space, time and noise (i.e. cellular compartmentalization, system dynamics and cell-cell variability) over the known system topologies. Instead, several groups have developed mathematical models for modules or pathways within the network, notably by modeling receptor trafficking, Ca^2+^ transients and ERK activation (Lim et al., 2009, Perrett et al., 2014, Stojilkovic et al., 2010, Stojilkovic, 2012, Washington et al., 2004). We have focused our attention on a simplified network encompassing the remarkably small group of chemicals acting on or within gonadotrophs that have been shown by knock-down or inactivating mutation to be essential for reproduction (namely GnRH, GnRHR, LH, FSH and ERK) and have added Ca^2+^ to this list in light of the wealth of evidence showing its requirement for hormone secretion (Fig. 1A). To explore this experimentally, we developed live cell imaging readouts based on nucleocytoplasmic translocation of ERK2-GFP, as a readout for activation of the Raf/MEK/ERK cascade, and of NFAT-EFP as a readout for Ca^2+^-dependent activation of the CaM/Cn/NFAT cascade (Armstrong et al., 2009a, Armstrong et al., 2010). As shown (Fig. 1B and C), pulses of GnRH cause nuclear translocation of both of these reporters: the ERK2-GFP translocation responses are rapid and transient whereas the NFAT-EFP responses are slower in onset and reversal. To develop mechanistic understanding we also constructed a deterministic mathematical model of GnRHR signaling that was trained on this wet-lab data and mirrors these ERK2-GFP and NFAT-EFP translocation responses (Tsaneva-Atanasova et al., 2012). More recently we developed a second model differing from the earlier version in three important respects; a) it is trained on data from signaling of endogenous GnRHR in LβT2 cells (rather than from signaling in Ad GnRHR-transduced HeLa cells), b) it is trained on full concentration-response curves (rather than just response dynamics at maximal GnRH concentrations, and c) it incorporates agonist-induced receptor internalization as an upstream negative feedback mechanism. A key feature of this model is that it includes compartmentalization (i.e. movement of components to and from the nucleus) as this is needed for training against wet-lab data for ERK2-GFP and NFAT-EFP translocation. This represents a vast oversimplification as other cellular structures are undoubtedly important for GnRH signaling and our current model could be modified directly to allow computational investigation of such compartments (Kholodenko et al., 2010, Neves et al., 2008, Neves and Iyengar, 2009). For example, spatial information could be included by consideration of the plasma membrane and lipid raft/plasma membrane microdomains (in addition to the cytosol and the nucleus) explicitly taking into account the area/volume of compartments, reactions occurring within them and associated fluxes to and from them. Nevertheless, we believe that the current model (given in the Supplemental Data) is a useful tool for exploring GnRH signaling. Fig. 2 shows data from simulations using the LβT2 cell trained model with 5 min square wave pulses of 10^−9^ M GnRH with 60 min period. Consistent with experimental data, the model predicts that each GnRH pulse will cause a pulse of receptor occupancy, PLC activation, cytoplasmic Ca^2+^ elevation and ERK activation. These are all rapid in onset and rapidly reversed on pulse termination. The Ca^2+^ and ppERK pulses are predicted to drive nuclear translocation of NFAT and activation of the ERK effector Egr1, both of which are relatively slow in onset and reversal (Fig. 2).

We have used this, and a similar model (Perrett et al., 2014), to explore system sensitivity to different input features, focussing on the ERK pathway with varied GnRH dynamics. This revealed, as expected, that increasing GnRH concentration 10-fold does not cause a 10-fold increase in responses, mainly because it does not increase GnRHR occupancy 10-fold. Moreover, increases in outputs caused by an x-fold increase in GnRH pulse width are less than the increases caused by an x-fold increase in pulse frequency. Thus, the system is an integrative tracker because it is sensitive to pulse amplitude, frequency and width (all of which influence the integral of the input), but there is certainly not a simple 1:1 relationship between integrated input and output. Instead, the kinetics of receptor occupancy and downstream effector activation create a system that is robust to changes in pulse width and concentration but sensitive to changes in pulse frequency, the input variable known to vary under different physiological conditions *in vivo* (Perrett et al., 2014).

**Fig. 3:**
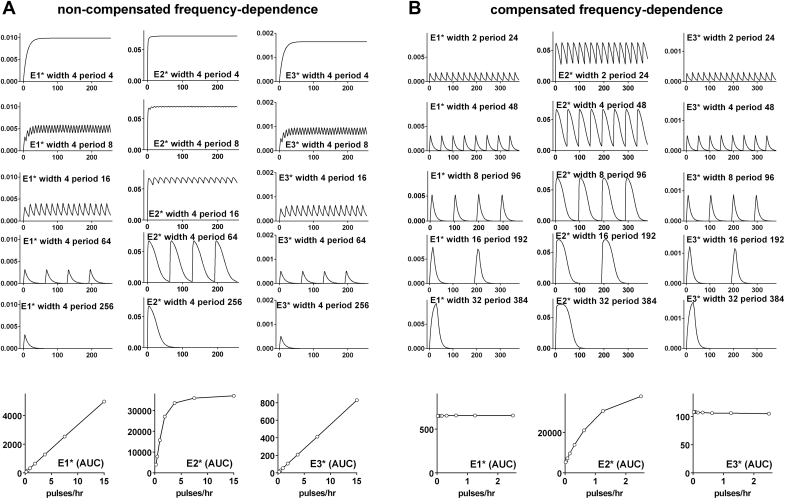
**Increasing efficiency and specificity of signaling with pulses: simulations with a minimal model**. We modelled activation of an effector E1, that in turn activates two downstream effectors, E2 and E3. The traces show active effector (E1*, E2* and E3* in arbitrary units) from simulations with square wave input pulse. Activation follows Michaelis-Menten type kinetics and parameters are set for rapid activation and inactivation of E1 and E3 and for slower activation and inactivation of E2 (see parameters in Supplemental Data). Fig. 3A shows simulations with a fixed pulse width of 4 min and varied pulse period (including constant stimulation with width and period both 4 min in the top row). In addition to the time-courses (top 5 rows) we show integrated outputs as area under the curve (AUC) for the activated effectors plotted against pulse frequency (bottom row). These are non-compensated frequency-response relationships where the input integral increases in direct proportion to the frequency. For comparison, Fig. 3B shows compensated pulsatile-stimulation where any increase in frequency is offset by a reduction in pulse width so that the input integral is identical for all frequencies. Note that for the non-compensated scenario, E1* and E3* AUCs are almost directly proportional to pulse frequency because responses are rapid in onset and reversal, but slower activation and inactivation causes a non-linear relationship between pulse frequency and E2* AUC. This effect is more obvious for the compensated scenario (Fig. 3B) where the rapid E1* and E3* responses again mirror the input integral and are therefore similar at all pulse frequencies, whereas for the slower E2* responses AUC increases with pulse frequency in spite of the fact that the integrated input is identical at all frequencies (i.e. the E1* and E3* plots are effectively flat lines whereas there is an increasing monotonic relationship for E2*). Fig. 3B therefore provides a simple illustration of an integrative tracking system with rapid outputs closely mirroring the integrated input and slower responses leading to a non-linear input-output relationship. This increases efficiency (multiple brief pulses cause greater output than single long pulses) and specificity (because the same change biases signaling toward E2* as compared to E3*).

We have taken a similar approach to address the question of why pulsatile inputs are so prevalent in biological systems. Here, the most obvious answer is that it can increase efficiency and this is illustrated by our NFAT-EFP translocation data. With GnRH pulses at 30 min intervals there is insufficient time for responses to return to pre-stimulation values between pulses (Fig. 1C, red line) so there is a cumulative (saw-tooth) response that is very close to the response obtained with constant stimulation (see also Fig.10.6 in (McArdle and Roberson, 2015)). To explore this more thoroughly we developed a minimal model with a pulsatile stimulus activating an effector (E1) which, in turn, activates two downstream effectors (E2 and E3) in parallel. We modelled this with Michaelis-Menten type kinetics with parameters chosen to elicit rapid activation and inactivation of E1 and E3 but much slower activation and inactivation of E2 (see model parameters in Supplemental Data). Fig. 3A shows simulations with a fixed pulse width of 4 min and varied pulse period from 4 to 256 min (note that the top row shows constant stimulation with width and period both 4 min). In addition to the time-courses (top 5 rows) we show integrated outputs as area under the curve (AUC) for the three activated effectors (E1*, E2* and E3*) plotted against pulse frequency (bottom row). As shown there is a near linear relationship between pulse frequency and E1* AUC because responses are rapid in onset and reversal and the same is true for E3* AUC because E3 is rapidly activated (by E1*) and inactivated. However, activation and inactivation of E2* are slower so signaling continues more beyond the stimulus pulse, a cumulative response occurs at lower period and there is a non-linear relationship between pulse frequency and E2* AUC. This effect is more obvious with a compensated frequency-response relationship. In this case any increase in pulse width is compensated for by a reduction in pulse frequency so that the input integral (i.e. the AUC for the pulsatile stimulus) is identical at all pulse frequencies (Fig. 3B), in contrast to the non-compensated frequency-dependence the input integral is directly proportional to pulse frequency (Fig. 3A). For the compensated inputs, the E1* AUC and E3* AUC values vary little with pulse frequency (Fig. 3B lower row) because responses are rapid and the system behaves as a simple integrative tracker, but for the E2* AUC increasing pulse frequency increases system output in spite of the fact that the integrated input is identical. From the lower row of Fig. 3B it is evident that the gradient of the E2* plot is >1, providing a clear demonstration of how efficiency can be increased by using a pulsatile input, and that the plots for E2* and E3* differ, demonstrating output specificity with pulsatile inputs. Thus, if we equate this to a neuroendocrine system with a finite amount of releasing hormone, system output (E2*) can be increased by using multiple brief pulses as compared to a single long pulse (compare width 2 period 24 with width 32 period 384) and this same change also biases signaling toward E2* (as compared to E3*).

**Fig. 4:**
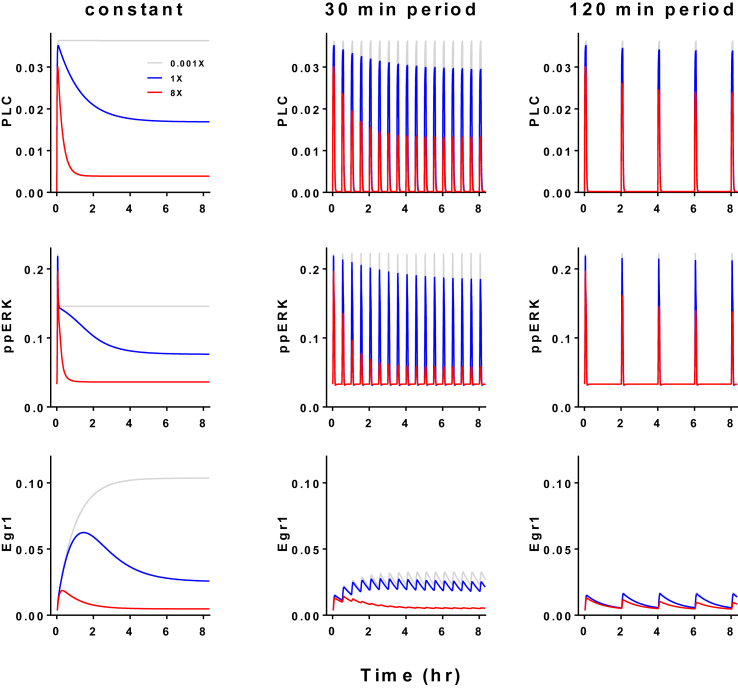
**Avoiding desensitization with pulses: simulations with an LβT2 cell-trained model**. The data shown are concentrations of active PLC, ppERK and Egr1 from simulations of responses to 10^−7^ M GnRH as a constant stimulus or as 5 min pulses at 30 or 120 min intervals as indicated. The model incorporates agonist-induced receptor internalization at a rate derived from fitting wet-lab data (1×) as well as at an extremely low rate (0.001×) and with an 8-fold increased rate (8×), as indicated. The data predict receptor internalization to have a pronounced effect with constant stimulation (compare grey and blue traces in column 1) but that its effect becomes increasingly negligible with pulsatile stimulation as period increases (compare grey and blue traces in columns 2 and 3).

Avoidance of desensitization is another often-cited reason for pulsatility in biological systems and we have explored this using the LβT2 cell-trained model. This incorporates agonist-induced GnRHR trafficking (internalization from and recycling to, the cell surface with parameters trained on radioligand binding data) and Fig. 4 shows simulations with 5 min GnRH pulses at varied period with all other parameters identical except that GnRHR internalization was set at 1×, 8× or 0.001× (as multiples of the estimate obtained from data and shown in Supplemental data table 1A). With constant stimulation (Fig. 4, left column) and negligible GnRHR internalization, PLC activity is predicted to increase rapidly to a sustained level but when receptor internalization is introduced there is an initial spike of PLC activity (within minutes) that reduces to a plateau (within hours). Similar effects occur downstream as all responses become smaller and/or more transient as the internalization rate increases. GnRHR internalization is also predicted to reduce responses with pulsatile GnRH (Fig. 4, right columns) but the effect is much less pronounced. Thus, for example, introduction of GnRHR internalization has a pronounced effect on PLC activity and ERK-dependent transcription (compare grey and blue traces in upper left and lower left plots) but has negligible effects at 120 min period (compare grey and blue traces in upper right and lower right plots) because internalization is driven by receptor occupancy which is clearly lower with pulsatile stimulation. These simulations were with 10^−7^ M GnRH whereas physiologically GnRH pulses are in the low nM range (McArdle and Roberson, 2015) so the data demonstrate that pulsatility mitigates the effect of GnRHR internalization and also emphasize the fact that pronounced agonist-induced down-regulation of cell surface GnRHR is more relevant to pharmacological stimulation than it is to physiological.

Extending the modeling outlined above, we simulated responses to 10^−7^ M GnRH as a constant stimulus or in pulses (5 min period 60 min interval), setting the internalization and recycling rates at 1× (again as multiples of the estimates obtained from data) or varying them by serial halving or doubling (i.e. from 0.03125× to 32×). Using the integrated PLC response as a readout we found, as expected, that with either paradigm increasing the rate of internalization reduced the response whereas increasing the rate of recycling increased it. With constant stimulation the system shows comparable sensitivity to internalization and recycling because they are equally important determinants of cell surface receptor number at equilibrium, and this is evidenced by the near symmetrical curves for internalization or recycling versus PLC activity in Supplemental Fig. 1A. However, with pulsatile GnRH the relationship between internalization rate and PLC activity is right shifted because agonist-induced internalization occurs only during the GnRH pulses so a greater increase in internalization is needed to achieve a given reduction in output. The system is more complex for recycling because of opposing tendencies; recycling can continue beyond the GnRH pulse and this tends to increase sensitivity to recycling whereas recycling applies only to the small proportion of receptors that have internalized and this tends to reduce sensitivity to changes in recycling rate. For the simulation parameters used here the nett effect was that pulsatile stimulation reduced sensitivity to the recycling rate (compare steepness of the filled circle plots in Supplemental Fig. 1A and B). When considering the physiological context, a particularly interesting feature of these simulations is that they predict near maximal system output with pulsatile stimulation and rates of internalization and recycling estimated from data. This contrasts to the markedly submaximal outputs with constant stimulation (as indicated by the double arrows in Supplemental Data Fig. 1) implying that the system has evolved for efficient receptor signaling with pulsatile stimulation.

**Fig. 5:**
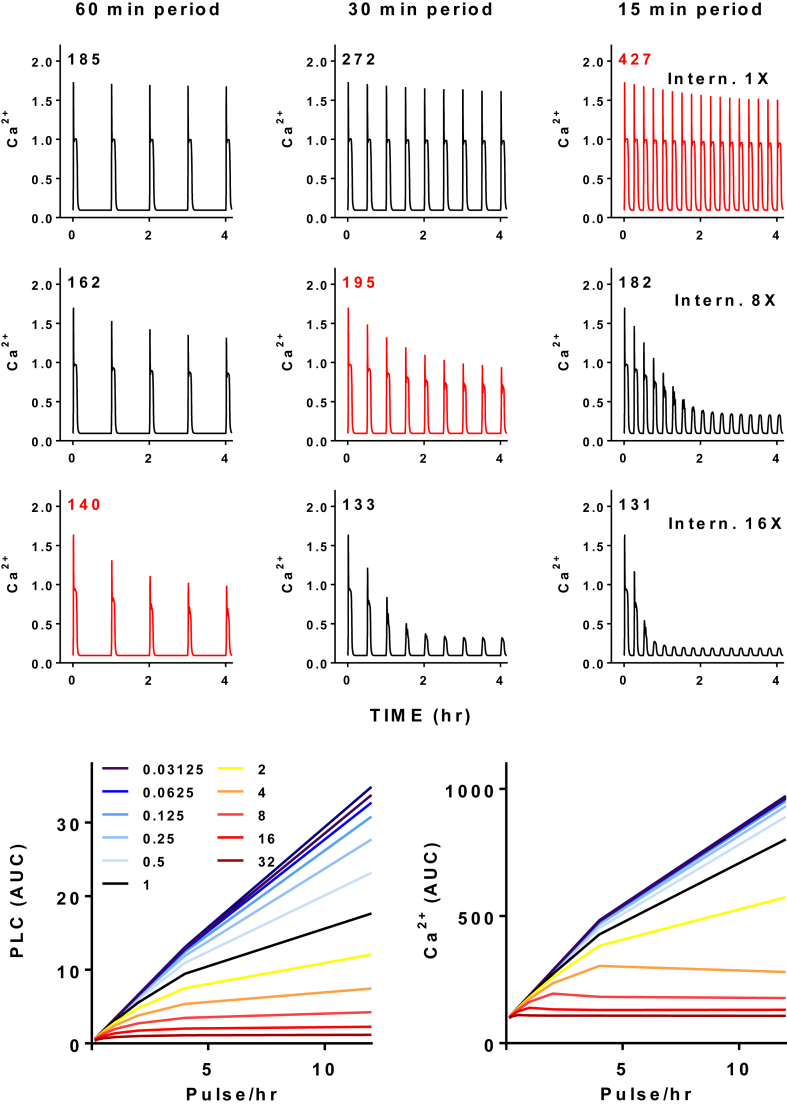
**Maximal output with sub-maximal inputs: simulations with varied feedback strength**. The upper three rows show simulated Ca^2+^ responses (μM cytoplasmic Ca^2+^ concentration) for the LβT2 cell-trained model using 5 min pulses of 10^−7^ M GnRH at 60, 30 or 15 min and incorporating upstream negative feedback as agonist-induced receptor internalization at a rate derived from fitting wet-lab data (1×) as well as at two increased rates (8× and 16×), as indicated. The AUC of the Ca^2+^ transients is calculated (for 960 min simulations) and for each GnRHR internalization rate the condition giving the highest Ca^2+^ AUC is shown in red. Note that as internalization rate is increased, pulse-frequency-dependent desensitization becomes more evident and, as a consequence of this the greatest output is achieved with sub-maximal GnRH pulse-frequency when GnRHR internalization is set at 8× or 16×. The bottom row shows GnRH pulse frequency-response relationships from a more extensive series of simulations with GnRHR internalization varied from 0.03125× to 32× and output AUCs shown for both active PLC and Ca^2+^. Note that maximal Ca^2+^ responses only occur at sub-maximal pulse frequency when GnRHR internalization rate is 4× or greater (i.e. where pronounced desensitization of Ca^2+^ responses occurs) and that the PLC responses are maximal with constant stimulation (i.e. 12 pulses of 5 min width per hour) for all GnRHR internalization rates.

Another fundamentally important feature of the GnRH signaling system is that responses can be maximal at sub-maximal pulse frequency (Ferris and Shupnik, 2006, Ciccone and Kaiser, 2009, Bedecarrats and Kaiser, 2003, Dalkin et al., 1989, Shupnik, 1990, Weiss et al., 1990, Kaiser et al., 1993, Haisenleder et al., 1991, Kanasaki et al., 2005, Ciccone et al., 2010). Moreover, the frequency eliciting maximal responses is dependent on the output, as seen in work with luciferase reporters for gonadotrope signature genes (Bedecarrats and Kaiser, 2003), where the optimal GnRH pulse frequencies for expression of LHβ, FSHβ, αGSU and GnRHR reporters differ (maximal responses at pulse intervals of 2 h for LHβ and FSHβ, 0.5 h for αGSU and 1 h for GnRHR, in LβT2 cells). The key observation here is that for many GnRH effects there is a non-monotonic (bell-shaped) pulse frequency-response curve. This could reflect the existence of feedback or feed-forward loops but the nature of these loops is unclear. Rapid homologous receptor desensitization can be excluded as a potential negative loop because type I mammalian GnRHR do not show this behavior (above). However, GnRH does down-regulate cell surface GnRHR and this alone could generate bell-shaped GnRH pulse frequency-response relationships as illustrated (for our LβT2 cell-trained model) in Fig. 5. The time-courses (Fig. 5 top 3 rows) show simulated Ca^2+^ responses with 5 min pulses of 10^−7^ M GnRH at varied period and at varied GnRHR internalization rates (1×, 8× and 16×, again as multiples of the estimate obtained from data). System output was calculated as the AUC for the Ca^2+^ concentration over 16 h and the condition giving the highest AUC is plotted in red (for each internalization rate). As shown, the system output was greatest at 15 min period with 1× internalization, at 30 min period with 8× internalization and at 60 min period with 16× internalization. Simulations with a broader range of pulse frequencies and internalization rates (Fig. 5, lower traces) revealed increasing monotonic frequency-response curves for GnRH effect on PLC at all internalization rates (from 0.03125× to 32×) and at most internalization rates for effects on Ca^2+^ but with GnRHR internalization at 4×, 8×, 16× and 32× maximal Ca^2+^ responses are predicted to occur at sub-maximal pulse frequency. These simulations therefore show how GnRHR internalization could generate non-monotonic frequency response relationships but only under conditions that are inconsistent with experimental data, with internalization rates, extent of receptor down-regulation and desensitization of Ca^2+^ responses much greater than seen experimentally. Alternative mechanisms for desensitization to GnRH have also been described and these include GnRHR-mediated induction of RGS (regulator of G-protein signaling)-2 (Karakoula et al., 2008), induction of MAPK phosphatases (Lim et al., 2009), down-regulation of IP_3_ receptors (Willars et al., 2001, Wojcikiewicz et al., 2003), and ERK-mediated negative feedback (Caunt et al., 2006, Armstrong et al., 2009a). However, such responses have been explored primarily with constant stimulation paradigms and may well have little effect with pulsatile stimulation. A thorough theoretical examination of pulse frequency decoding mechanisms also revealed how receptor dimerization can generate non-monotonic frequency-response relationships (Fletcher et al., 2014) and this is of particular interest in light of early studies suggesting that dimerization of GnRHR could elicit signaling (Conn et al., 1987, Conn et al., 1982), as well as work showing that agonists (but not antagonists) bring GnRHR closer to one-another (Navratil et al., 2006, Cornea et al., 2001) but it is not established that dimerization of normal GnRHR is a prerequisite for signaling. The live cell imaging experiments described above also provide some insight here, as the ERK2-GFP and NFAT-EFP translocation responses were both reproducible with repeated GnRH pulses (Fig. 1) and the signals passing from the cytoplasm to the nucleus showed increasing monotonic frequency-response relationships. In support of this, Egr1-responsive and NFAT-responsive luciferase reporters used as transcriptional readouts for ERK and NFAT activation both show maximal responses at maximal GnRH pulse frequency (Armstrong et al., 2009a, Armstrong et al., 2009b, Armstrong et al., 2010).

Taken together, the work outlined above shows how upstream negative feedback could theoretically generate bell-shaped frequency response relationships but also suggest that such feedback is insufficient to shape GnRH signaling with physiologically relevant pulsatile stimulation. Where signaling inputs to the nucleus show increasing monotonic frequency-response relationships, the obvious possibility is that feedback and/or feed-forward regulatory loops within the nucleus underlie the observed bell-shaped frequency-response relationships for gene expression. This has been explored most extensively for the FSHβ promoter, for which a number of incoherent feed-forward loops have been described. These are signaling modules that fan out from an upstream node and re-converge at a downstream node and for which the two divergent branches have different overall signs (i.e. positive and negative effects). Thus, for example, stimulation of FSHβ gene expression by GnRH is, in part, mediated by its ability to phosphorylate and activate the transcription factor CREB, but GnRH can also increase expression of the inducible cAMP early repressor (ICER), which inhibits the effect of CREB, providing both positive and negative inputs to the promoter (Ciccone et al., 2010, Thompson et al., 2013). As noted above, pulsatile stimulation provides the potential for specificity in effector activation and the inhibitory (ICER-mediated) loop is preferentially activated at high GnRH pulse frequency so that transcriptional activation is greatest at sub-maximal pulse frequency. Similarly, it was shown that expression of Fos and Jun (positive regulators of FSHβ expression) is increased at lower GnRH pulse frequencies than needed for expression of negative regulators (the co-repressors SKIL, CREM and TGIF1) suggesting regulation by an alternative incoherent feed-forward loop in which SKIL and/or TGIF1 inhibit activation by AP-1 factors Fos and Jun (Mistry et al., 2011). In addition to these nuclear mechanisms, incoherent feed-forward loops have been described in which the inhibitory branch is due to GnRH-stimulated protein secretion. In the first, it is mediated by secretion of inhibin-α, which has long been known to supress FSH expression, and in the second it is mediated by inhibition of the secretion of growth differentiation factor 9, an autocrine inducer of FSHβ expression in LβT2 cells (Choi et al., 2012, Choi et al., 2014, Pincas et al., 2014).

**Fig. 6:**
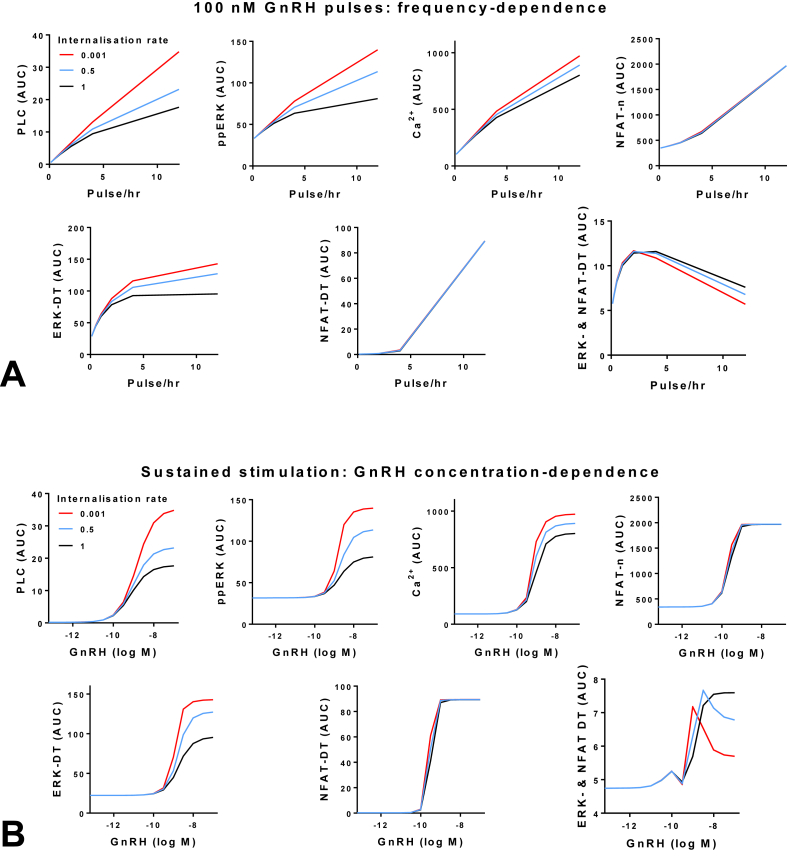
**Maximal output with sub-maximal input: simulations with co-operative convergent regulation of gene expression**. The LβT2 cell-trained model was used to simulate GnRH signaling at various levels in the GnRHR network (PLC activity, nuclear ppERK, cytoplasmic Ca^2+^, nuclear NFAT) and also for ERK-driven transcription (ERK-DT), NFAT-driven transcription (NFAT-DT) and the situation where ERK and NFAT converge and act co-operatively to drive transcription (ERK- & NFAT-DT) as described (Tsaneva-Atanasova et al., 2012). Panel A shows output AUCs for 960 min simulations with 5 min pulses of 10^−7^ M GnRH at varied frequency (including constant stimulation with 12 pulses/hr) and with GnRHR internalization at a rate derived from fitting wet-lab data (1×) as well as at negligible or low rates (0.001× and 0.5×). Note that for all conditions increasing monotonic frequency-response curves are obtained except for the ERK- & NFAT-DT, for which bell-shaped frequency-response relationships are seen, even with negligible negative feedback (Fig. 6A, lower right). Panel B shows data from simulations with constant stimulation at varied GnRH concentration. As shown, increasing monotonic concentration-response curves are obtained for all outputs except for ERK- & NFAT-DT where maximal responses are predicted for sub-maximal GnRH concentration when GnRHR internalization is at 0.5× or 0.001×.

We have also used mathematical modeling to explore possible frequency decoding involving the Raf/MEK/ERK and CaM/Cn/NFAT pathways as inputs to the transcriptome. We assumed that two transcription factors (i.e. NFAT and an undefined ERK-dependent transcription factor) act at separate sites on a common gene promoter and considered three different logic gates; an “and-gate”, an “or-gate” or a “co-operative gate”. This model predicted bell-shaped frequency-response relationships when two transcription factors act co-operatively. The characteristic feature of maximal response at sub-maximal frequency was never seen with the and-gate or with the or-gate, and this behavior was predicted without negative feedback (Tsaneva-Atanasova et al., 2012). More recently, similar simulations were run using our LβT2 cell-trained determinist model (Supplemental Data), again with 5 min pulses of 10^−7^ M GnRH at varied period and with varied GnRHR internalization rates. Fig. 6A shows predicted frequency response relationships for GnRH effects on PLC, nuclear ppERK, cytoplasmic Ca^2+^ and nuclear NFAT as well as predicted transcriptional responses driven by ERK or NFAT alone (ERK-DT and NFAT-DT) and in each case maximal system outputs are predicted at maximal pulse frequency. However, simulations assuming co-operative convergence of the two transcription factors at a promoter reveals non-monotonic frequency–response relationships at all three internalization rates (i.e. non-monotonic relationships due to co-operative convergence at the transcriptome rather than due to negative feedback). Interestingly, when the same parameters were used to explore GnRH concentration-dependence (with constant, rather than pulsatile GnRH) the simulations suggest that GnRHR internalization influences the balance of signaling via ERK and NFAT (i.e. the red and black lines in Fig. 6B differ markedly for ERK-DT but not for NFAT-DT) and most importantly, that the co-operative convergent model predicts non-monotonic concentration response curves with low GnRHR internalization rates. This modeling clearly does not show that the bell-shaped frequency-response relationships seen for transcriptional effects of GnRH are mediated by convergence of NFAT and ERK-dependent transcription factors because, in reality multiple pathways converge to mediate GnRH effects on transcription (Nelson et al., 1998). Moreover, the relative importance and mechanisms of integration of these inputs is undoubtedly promoter/enhancer-specific and the mathematical description of co-operative convergence is essentially a coherent feed-forward loop for which biological substrates have not been identified.

## 5 GnRH signaling: an information theoretic approach

Biological experiments are often undertaken assuming that all cells of a given “type” are identical, but numerous studies have shown that individual cells in a population differ quite markedly. In fact such cell-to-cell variation is inevitable because the processes underpinning cell behavior are stochastic. Most importantly, these differences can drive the health and function of the cell population because it is individual cells that have to sense their environment and make appropriate decisions (to express or suppress given genes, to survive or die, to proliferate or differentiate etc.) in light of it. The simulations outlined above effectively model the behavior of a typical GnRH-stimulated cell as representative of the whole population and ignore the cell-to-cell variation that has already been documented for GnRH effects on cytoplasmic Ca^2+^ concentration, gonadotropin secretion, effector activation and gene expression (Armstrong et al., 2009a, Armstrong et al., 2009b, Armstrong et al., 2010, Armstrong et al., 2009b, Lewis et al., 1989, Stojilkovic and Catt, 1995, McArdle et al., 1992, Ruf et al., 2006, Ruf et al., 2007, Caunt et al., 2012, Garner et al., 2016).

Information theory was developed to analyze electronic communication but is now also being used to measure how reliably biological signaling systems transfer environmental information (Cheong et al., 2011, Brennan et al., 2012, Voliotis et al., 2014, Bowsher et al., 2013, Bowsher and Swain, 2014, Uda et al., 2013, Selimkhanov et al., 2014). Here, ‘information’ is taken to mean the uncertainty about the environment that is reduced by signaling, and can be quantified using Mutual information (MI), a statistical measure of the quality of inference of the signal from the cellular response (Bowsher and Swain, 2014). MI is measured in Bits with an MI of 1 Bit meaning that the system can unambiguously distinguish between two equally probable states of the environment. For cell signaling studies, the signal could be the concentration of stimulus and the response could be the amount of activated effector in an individual cell. Where information theoretic approaches are used to analyze cell signaling, the signaling pathways are effectively treated as noisy communication channels and MI is used as measure of the amount of information that they carry. Key points here are that instead of ignoring cell-to-cell variation this approach considers how it influences information transfer, and that instead of focussing on identification of signaling intermediates in a pathway, this approach seeks to quantify the amount of information that the pathway transfers or could transfer.

**Fig. 7:**
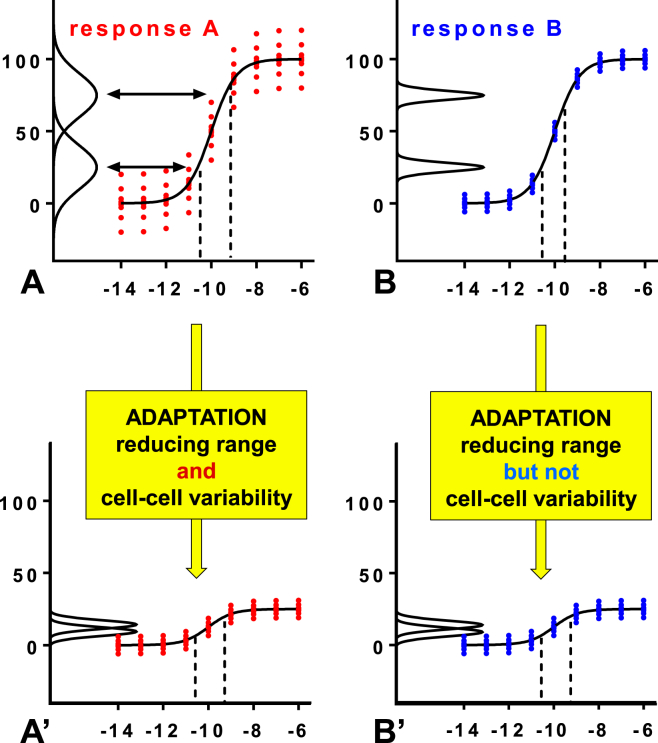
**Cell-cell variability and information transfer**. The solid sigmoid curves in the upper cartoons illustrate population averaged responses, with individual dots representing single cell responses from which the population averages are derived. For panels A and B the population averaged data are identical but there is higher cell-cell variability in A. Consequently, frequency distribution plots shown on the left (for the stimulus concentrations indicated by the dotted lines) overlap for panel A. This creates a region of uncertainty, in that any individual cell in the area of overlap cannot “know” which stimulus concentration it has been exposed to. For panel B, cell-cell variability is much lower so the frequency-distributions do not overlap and there is no area of uncertainty. Mutual information is a statistical measure of inference quality (how reliably the system input can be inferred from the output). It is measured in Bits (with an MI of 1 indicating a system that can unambiguously distinguish two equally probable states of the environment) and would be higher in B than in A. We also illustrate the situation where the cells adapt to their environment such that the population averaged response is reduced either with a proportional reduction in cell-cell variability (A→A′) or with no change in cell-cell variability (B→B′). Note that the frequency-distributions overlap in A′ just as they do in A, and in B′ whereas they don't in B. Accordingly, the B→B′ adaptive response reduces information transfer whereas the A→A′ adaptation does not. In this scenario, consideration of population averaged responses alone can clearly deliver the wrong conclusion; if this were a hormone pre-treatment protocol one would conclude that the system has desensitized from A to A′ in spite of the fact that the quality of hormone sensing has not altered.

The value of this approach can be illustrated by considering a simple signaling network that bifurcates and adapts over time as shown in Fig. 7. For effectors A and B the population averaged input-output relationships are identical (panels A and B) but there is higher cell-to-cell variability for A than for B as illustrated by the broader spread of red dots (representing individual cells) and the frequency-distribution plots (black lines on y-axis). For the two stimulus concentrations shown by the dotted lines and arrows, it is evident that the frequency distribution plots overlap for A but not for B. Accordingly, there is a region of uncertainty with individual cells in A being unable to unambiguously distinguish these two states of the environment whereas all individual cell in B can do so. Thus, the quality of the inference of the signal from the response is lower for A than for B (i.e. the MI between B and the signal is greater than the MI between A and the signal). We now assume that the system incorporates negative feedback loops and adapts over time so that the population averaged outputs are reduced and again, the population averaged responses are identical for the adapted (desensitized) system (compare black lines in A′ and B′). However, negative feedback has the potential not only to reduce the population averaged response but also to reduce cell-cell variability. For the A→A′ adaption we assume that cell-to-cell variability and population averaged response reduce in parallel so that the overlap between the frequency distribution plots remains (albeit scaled) so that the quality of sensing is not actually reduced. In contrast, we assume that for the B→B′ transition the population averaged response reduces without a reduction in cell-to-cell variability so the frequency-distribution plots overlap for the adapted system and the quality of sensing is reduced. Here, it is evident that consideration of the population averaged response alone can deliver the wrong conclusion because the population averaged data show that the system has clearly desensitized from A to A′ yet the reliability with which cells sense the stimulus has not. Moreover, consideration of population-averaged data alone suggests that balance of signaling to A and B is unaltered by adaptation yet this scenario shows that information transfer to A is less than is to B, and that this imbalance is lost after adaptation. More generally, we have used a stochastic model to explore information transfer through a kinase cascade and showed how negative feedback can reduce sensing (by reducing the response dynamic range) or improve sensing (by reducing cell-cell variability) and that the independent regulation of these effects means that population averaged responses do not provide reliable measures of information transfer (Garner et al., 2016).

**Fig. 8:**
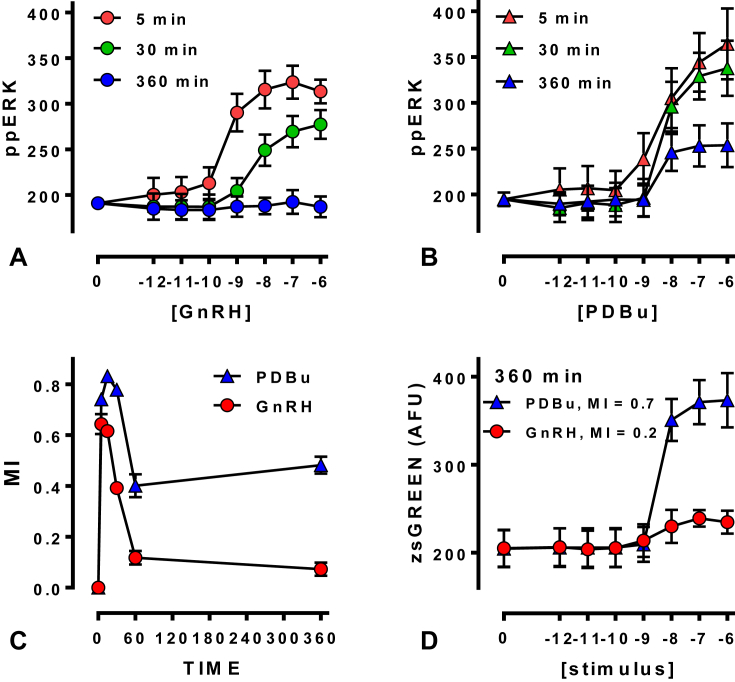
**MI as an information theoretic measure of GnRH sensing**. Panels A and B show concentration and time-dependent effects of GnRH and PDBu on ERK activity in LβT2 cells, with nuclear ppERK values measured by automated fluorescence microscopy and reported in arbitrary fluorescence units (AFU, mean ± SEM, n = 3–4). The single cell measures underlying these plots were also used to calculate MI between ppERK and each of these stimuli and these values are plotted (I(ppERK; stimulus) in Bits) against time in panel C. These cells were also transduced with recombinant adenovirus for expression of an ERK-driven transcription reporter (Egr1-zsGREEN). Panel D shows the concentration-dependence of GnRH and PDBu on zsGREEN expression (in AFU, mean ± SEM, n = 3) after 360 min stimulation and the MI between zsGREEN and each of these stimuli is also shown for this time. Adapted from Garner et al., (2016).

We recently used this approach to explore information transfer in HeLa cells that were transduced with recombinant adenovirus for GnRHR expression before stimulation for varied times and with different concentrations of GnRH. ppERK and nuclear translocation of NFAT-EFP were used as activation readouts, and Egr1- or NFAT response element-driven fluorophore expression were used as readouts for transcription activation by ERK and NFAT. Responses were measured in large numbers of individual GnRH-stimulated cells (Garner et al., 2016) and used to calculate MI between GnRH concentration and ppERK (I(ppERK; GnRH)). This revealed information transfer between GnRHR and ERK to be <1 Bit (Fig. 8). This is comparable to values obtained for cytokine and growth factor signaling in other systems but is still surprisingly low for two reasons. First, the cells were typically stimulated with eight concentrations of GnRH so there was a 3 Bit input (log_2_8), of which <1 Bit of information was transferred. Second, population-averaged measures consistently show responses to GnRH being graded over a wide range of GnRH concentrations, yet an MI of <1 implies that single cells cannot unambiguously distinguish between just two inputs (i.e. with and without GnRH). This was not due to use of a heterologous expression system because information transfer values were similar in HeLa cells (with exogenous GnRHR) and LβT2 gonadotropes (with endogenous GnRHR). It was also not restricted to the ERK pathway because information transfer from GnRHR to NFAT was <0.5 Bits in both cell models (Garner et al., 2016). Another possible explanation for low information transfer is that single time-point measures underestimate information transfer. This would be expected where cells infer inputs (i.e. GnRH concentrations) from trajectories of outputs (i.e. ppERK levels) over time (Selimkhanov et al., 2014). For example, time-course experiments revealed that I(ppERK; GnRH) is higher at 5 than at 360 min (Fig. 8) but this clearly does not mean that a cell obtains less information over 360 min than it does over 5 min. Instead, it shows that the 360 min snapshot underestimates information transferred over the 360 min stimulation. Measuring MI for ERK-driven transcription is an alternative approach that could be sensitive to ppERK trajectory and, consistent with this, work with imaging readouts for ERK-driven transcription revealed more reliable sensing of PDBu than of GnRH in HeLa cells (Fig. 8), presumably because PDBu has a more sustained effect than GnRH on ppERK and causes a more marked increase in Egr1-driven zsGREEN expression (Garner et al., 2016). Thus the system senses sustained stimulation more reliably and must therefore be sensitive to the dynamics of ERK activation. This information theoretic approach was also applied to consider possible effects of negative feedback, focussing on ERK-dependent feedback (i.e. rapid transcription-independent and slow transcription-dependent feedback) and on receptor desensitization (i.e. by comparison of type I mammalian GnRHR that do not rapidly desensitize and XGnRHR that do). The overriding observation from these first statistical measures of information transfer via GnRHR is that it is not measurably influenced by the occurrence or absence of rapid receptor desensitization, but is influenced by downstream adaptive processes (i.e. ERK mediated feedback) with optimal GnRH sensing at intermediate feedback intensities.

## 6 Summary

Since GnRH was isolated and sequenced in the 1970s there have been immense advances in our understanding of GnRH signaling and our ever-increasingly complex GnRHR signaling networks highlight the necessity for mathematical and statistical analyses. The occurrence of maximal GnRH effects at sub-maximal GnRH pulse frequency is a fundamental and physiologically important feature of GnRH signaling that has still not been adequately explained. The literature contains evidence that this is due to a) upstream negative feedback b) co-operative convergence of distinct pathways and c) the existence of incoherent feedforward loops. Our mathematical modeling argues against (a) as it requires strong negative feedback and associated pronounced desensitization that is not evident with our pulsatile stimulation paradigms (Fig. 5). Indeed, it seems likely that pulsatile GnRH secretion and the resistance of type I mammalian GnRHR to desensitization both serve to minimize negative feedback and thereby place increasing reliance on alternative mechanisms. The second stems primarily from our mechanistic modeling. Its main limitations are that the mathematical description of convergence used is one for which biological substrates have not been identified, and that simulations often reveal bell-shaped concentration-response curves whereas most wet-lab data for constant stimulation does not (Fig. 6). The third invokes incoherent feed-forward loops for which biological substrates are known but, to our knowledge, have not been mathematically modelled. A key question here is whether or not incoherent feed-forward loops that certainly can generate non-monotonic dose-response relationships (Alon, 2007, Mangan and Alon, 2003) also generate bell-shaped frequency-response relationships and indeed, whether there is a biologically meaningful parameter space in which GnRH pulses would drive bell-shaped frequency-response relationships and increasing monotonic dose-responses. This is an area that we are actively exploring *in silico* and experimentally.

## The abbreviations used are

GnRH: gonadotropin-releasing hormone, with –I, –II or –III where a specific form is meant, or without suffix as common usage for GnRH-I
GnRHR: GnRH receptor
LHRH: luteinizing hormone-releasing hormone
LH: luteinizing hormone
FSH: follicle-stimulating hormone
GSU: gonadotropin subunit
PLC: phospholipase C
IP_3_: inositol 1,4,5 trisphosphate
DAG: diacylglycerol
PKC: protein kinase C
MAPK: mitogen-activated protein kinase
ERK: extracellular signal regulated protein kinase, used here to mean ERK1 and/or ERK2 unless specific suffix is given
ppERK: ERK with dual phosphorylation in the TEY activation loop
MEK: MAPK/ERK kinase
JNK: Jun n-terminal kinase
CaM: calmodulin
Cn: calcineurin
NFAT: nuclear factor of activated T-cells
NFAT-RE: NFAT response element
NFAT-DT: NFAT-driven transcription
ERK-DT: ERK-driven transcription
GFP: green fluorescent protein
EFP: emerald green fluorescent protein
cAMP: cyclic adenosine monophosphate
CREB: cAMP response element binding protein
ICER: inducible cAMP early repressor
PDBu: phorbol 12, 13-dibutyrate
EGF: epidermal growth factor
