# Supplementary material for: Mathematical modeling of gonadotropin-releasing hormone signaling
Source: Mol Cell Endocrinol. 2017 Jul 5;449:42–55. doi: 10.1016/j.mce.2016.08.022 (PMC5446263; doi:10.1016/j.mce.2016.08.022)
Supplement: Supplementary file 1 [file mmc1.docx]

**Supplemental Data**

**Mathematical Modeling of Gonadotropin-Releasing Hormone Signaling**

Amitesh Pratap, Kathryn L Garner, Margaritis Voliotis, Krasimira Tsaneva-Atanasova and

Craig A McArdle

**1. LβT2 *in-silico* model.**

A set of deterministic Ordinary Differential Equations (ODEs) was used to model the $\text{NFAT}$ (nuclear factor of activated T cells) and $\text{ERK}$ (extracellular signal regulated kinase) signaling pathways in LβT2 cells as described in the main text. Here we present the mathematical equations and the associated kinetic parameters of our LβT2 model. This is a modification of an earlier *in silico* model that was based on data for GnRHR signaling in HeLa cells (Tsaneva-Atanasova, Mina, Caunt et al., 2012).

The pulsatile$\text{GnRH}$signal is modelled as a sequence of square-shaped pulses using a Heaviside step function$\Theta$as follows,

$$GnRH=p(\Theta(\mathrm{mod}(t,T))-\Theta(\mathrm{mod}(t,T)-t_{p})).$$

The equation above describes time-evolution of $\text{GnRH}$*pulse-train* of amplitude $\text{p}$, time period $\text{T}$and pulse duration $\text{t}_{\text{p}}$. The ODEs model the $\text{GnRH}$ binding to the $\text{G}$protein-coupled receptor $\text{GnRHR}$ (denoted by $\text{R}$) to form hormone receptor complex $\text{HR}$resulting in the activation/release of the$\text{G}^{\text{*}}_{\text{aq/11}}$ protein from $\text{GnRHR}$,

$$\frac{d[HR]}{dt}=k_{1}[GnRH]([R_{0}]-[R_{fin}]-[HR])-k_{-1}[HR]-k_{2}([G_{0}]-[G^{*}_{\alpha q/11}])[HR]+k_{-2}[G^{*}_{\alpha q/11}]$$

$$\frac{d[G^{*}_{\alpha q/11}]}{dt}=k_{2}([G_{0}]-[G^{*}_{\alpha q/11}])[HR]-k_{-2}[G^{*}_{\alpha q/11}]$$

The asterisks denote the active form of the species. The negative feedback on the receptor-hormone binding caused by the receptor internalization is modelled using the following two differential equations

$$\frac{d[R_{mid}]}{dt}=k_{3intmid}[HR]\frac{[R_{0mid}]-[R_{mid}]}{Km_{1intmid}+[R_{0mid}]-[R_{mid}]}-k_{4intmid}\frac{[R_{mid}]}{Km_{2intmid}+[R_{mid}]}$$

$$\frac{d[R_{fin}]}{dt}=k_{3int}\frac{[R_{mid}]([HR])^{n_{int}}}{([Km1_{int}])^{n_{int}}+([HR])^{n_{int}}}-k_{4int}[R_{fin}],$$

We assume that the internalization is coordinated by the intermediate effector$\text{R}_{\text{mid}}$, which after being activated by the hormone-receptor complex $\text{HR}$mediates formation of the receptor internalization complex $R_{fin}$. The complex then internalises and recycles back to the membrane at the rates described by parameters $\text{k}_{\text{3int}}$ and $\text{k}_{\text{4int}}$respectively.

The activation of $\text{PLC}$ by $\text{G}^{\text{*}}_{\text{aq/11}}$ is described using Michaelis-Menten type kinetics,

$\frac{d[PLC^{*}]}{dt}=k_{e2}[G^{*}_{\alpha q/11}]\frac{[PLC_{0}]-[PLC^{*}]}{[PLC_{0}]-[PLC^{*}]+K_{Ee}}-k_{-e2}[PLC^{*}]$.

The hydrolysis of$\text{PIP2}$by$\text{PLC}^{\text{*}}$, to produce the secondary messenger$\text{IP3}$, is described by the following equation

$$\frac{d[IP3]}{dt}=k_{4}[PLC^{*}]-k_{-4}[IP3].$$

The Hodgkin-Huxley type model (Hodgkin and Huxley, 1952), which describes$\text{IP3}$mediated Ca^2+^ release from the endoplasmic reticulum (ER) via$\text{IP3R}$channels, was taken from earlier work (Li and Rinzel, 1994). The model was simplified by assuming independence of the three binding reactions involved in the gating of the channel to obtain the following equations

$$\frac{d[Ca^{2+}]}{dt}=\frac{fi}{Vi}\left(L+P\left(\frac{[IP3][Ca^{2+}]h}{([IP3]+Ki)([Ca^{2+}]+Ka)}\right)^{3}\left(\frac{[Ca_{0}^{2+}]-[Ca^{2+}]}{\sigma}-[Ca^{2+}]\right)\right.$$

The equation above describes the evolution of the cytosolic Ca^2+^ concentration $\text{[Ca}^{\text{2+}}\text{]}$. The variable $\text{h}$represents the fraction of channels not yet inactivated by Ca^2+^. In order to take into account the fast activation and slow deactivation of the $\text{IP3R}$ channels, as has been observed experimentally, steady state expressions were used for the activation of the channel, given by

$\frac{[IP3]}{([IP3]+Ki)}\frac{[Ca^{2+}]}{([Ca^{2+}+Ka)}$.

The parameters $\text{V}_{\text{e}}$ and $\text{K}_{\text{e}}$ describe the rate kinetics and the Ca^2+^ affinity of the SERCA (sarcoplasmic and endoplasmic reticulum ATPase) pumps, which pump cytosolic Ca^2+^ back into the ER. Similarly, the parameters $\text{V}_{\text{p}}$, $\text{K}_{\text{p}}$ determine the kinetics of Ca^2+^ export from the cytosol across the plasma membrane to the extracellular medium, whereas $\text{J}_{\text{in}}$represents the rate of Ca^2+^ flux from the extracellular medium. The total amount of Ca^2+^ $\text{[Ca}_{0}^{\text{2+}}\text{]}$ in the cell is only affected by the exchange of Ca^2+^ from the extracellular media and was modelled as the following,

while the rate of change of the fraction of deactivated channels is described by,

$$\frac{dh}{dt}=A(K_{d}-(([Ca^{2+}]+K_{d})h)).$$

**1.1. NFAT nuclear translocation.** In the following we outline the ODEs describing the$\text{NFAT}$nuclear cycle, in which $\text{NFAT}$is dephosphorylated to facilitate nuclear import, and then rephosphorylated to facilitate nuclear export. This modelling closely follows that of Cooling et al. (Cooling, Hunter and Crampin, 2009). Here, Ca^2+^ binds to and activated calmodulin which in turn activates the protein phosphatase calcineurin. The activation of calcineurin by calcium-bound calmodulin is assumed to be fast, which gives the following Hill type expression for the fraction of the activated calcineurin for a given concentration of cytosolic Ca^2+^,

$$act_{N}=\frac{[Ca^{2+}]^{n}}{[Ca^{2+}]^{n}+k_{mn}^{n}(1+(k_{d1}/M))}.$$

At a given concentration of calmodulin $\text{M}$, $\text{k}_{\text{m,N}}$is the half-maximal activation concentration of Ca^2+^ for the activation of calmodulin. Parameter $\text{k}_{\text{d1}}$is calcineurin-calmodulin disassociation constant. In the following subscripts$\text{c}$and $\text{n}$respectively indicate whether the protein is located in the cytosol or the nucleus. Combining the rate equation while adjusting for the difference in the volumes of the nucleus and cytosol using the dimensionless factor $\text{C}_{\text{cn}}$ (ratio of the two volumes), the following ODEs were used describe the $\text{NFAT}$cycle,

$$\frac{d[NFAT_{c}]}{dt}=J_{1}-J_{2}+l_{dp}[NFATp_{c}],$$

$$\frac{d[NFAT_{n}]}{dt}=J_{2}C_{cn}-J_{3},$$

$$\frac{d[NFATp_{c}]}{dt}=J_{2}C_{cn}-J_{3}-l_{dp}[NFATp_{c}],$$

$$\frac{d[NFATp_{n}]}{dt}=J_{3}-J_{4}.$$

Where

$$\begin{matrix} J_{1}=k_{f1}N_{tot}[NFATp_{c}]act_{N}-k_{r1}[NFATN_{c}](1-act_{N}), & J_{2}=[NFATN_{c}]k_{f2} \end{matrix}$$

$$\begin{matrix} J_{3}=k_{f3}[NFATN_{n}](1-act_{N})-k_{r3}N_{tot}[NFATNp_{n}]act_{N}, & J_{4}=[NFATNp_{n}]k_{f4}. \end{matrix}$$

Here$\text{N}_{\text{tot}}$, $\text{NFATp}_{\text{c}}$and $\text{NFATN}_{\text{c}}$ are the total amount of calcineurin, phosphorylated cytosolic $\text{NFAT}$and non-phosphorylated cytosolic$\text{NFAT}$bound to active calcineurin respectively. The term $\text{l}_{\text{dp}}$determines the basal rate of dephosphorylation of the cytosolic$\text{NFAT}$.

**1.2. ERK nuclear translocation.** The$\text{PLC}^{\text{*}}$initiated phosphorylation of $\text{MEK}$(mitogen-activated protein kinase/ERK kinase) leading to the phosphorylation of $\text{ERK}$ involves multiple steps. In the model the steps involved in phosphorylation cascade are assumed to be fast compared to the other time-scales. Thus the concentration of the active $\text{MEK}^{\text{*}}$for a given$\text{PLC}^{\text{*}}$concentration is simply given by the steady state expression,

$$[MEK^{*}]=\frac{k_{MEK}[MEK_{0}][PLC^{*}]}{(K_{de}+[PLC^{*}])}.$$

The $\text{ERK}$dynamics in the following describes the $\text{MEK}^{\text{*}}$mediated dual phosphorylation of $\text{ERK}$in the cytosol, nuclear import of the phosphorylated $\text{ERK}$, the nuclear dephosphorylation and the subsequent export of the $\text{ERK}$to the cytosol. The ODEs used to describe phosphorylation and dephosphorylation $\text{ERK}$ are modified from earlier modelling (Markevich, Hoek and Kholodenko, 2004). The phosphorylation, dephosphorylation of the cytosolic $\text{ERK}$and its translocation to and from the nucleus is given by

$$\frac{d[E_{c}]}{dt}=v_{4}-v_{1}+J_{expE}/C_{cn}-l_{imp}[E_{c}], J_{expE}=k_{expE}[E_{n}]$$

where $\text{v}_{4}$and $\text{v}_{1}$ are rates of dephosphorylation for singly phosphorylated $\text{ERK}$ $\text{E}_{\text{p}}$ and phosphorylation of unphosphorylated $\text{E}_{\text{c}}$ respectively. $\text{J}_{\text{expE}}$is the rate of export of the unphosphorylated $\text{E}_{\text{c}}$from the nucleus. A leaky import term described by the rate $\text{l}_{\text{imp}}$ has been added to account of basal trafficking of unphosphorylated $\text{E}_{\text{c}}$ into the nucleus. Similarly, the import of the dually phosphorylated $\text{ERK}$from the cytosol to the nucleus is described by $\text{J}_{\text{impE}}$

$$\frac{d[Epp_{c}]}{dt}=v_{2}-v_{3}-J_{impE}+l_{bas}+l_{exp}[Epp_{n}]/C_{cn}, J_{impE}=k_{impE}[Epp_{c}].$$

Here, $\text{v}_{2}$and $\text{v}_{1}$ are rates of phosphorylation of $\text{Ep}$ and dephosphorylation of $\text{Epp}_{\text{c}}$ respectively. A leaky export term here describes the basal rate $\text{l}_{\text{exp}}$ of trafficking of the dually phosphorylated $\text{Epp}_{\text{n}}$ from the nucleus to the cytosol. The rates of change of the concentrations of dually phosphorylated $\text{[Epp}_{\text{n}}\text{]}$, and unphosphorylated $\text{E}_{\text{n}}$ in the nucleus are dictated by the dephosphorylation rate $\text{v}_{5}$, and other export and import rates, and are expressed as

$$\frac{d[E_{n}]}{dt}=v_{5}-J_{expE}+l_{imp}[E_{c}]C_{cn}$$

$$\frac{d[Epp_{n}]}{dt}=J_{impE}C_{cn}-v_{5}-l_{exp}[Epp_{n}]$$

We note from above that, unlike in the cytosol, dephosphorylation of dually phosphorylated nuclear $\text{Epp}_{\text{n}}$is assumed dephosphorylate from$\text{Epp}_{\text{n}}$directly into $\text{E}_{\text{n}}$. Similar to $\text{NFAT}$dynamics, the dimensionless factor $\text{C}_{\text{cn}}$accounts for the dilution of the $\text{ERK}$once it has exited the nucleus. For completeness we include all the mathematical expressions for the various phosphorylation and dephosphorylation rates are shown below. Further details of the kinetic constants involved in the dual distributive phosphorylation of $\text{ERK}$can be found in (Markevich et al., 2004).

$$v_{4}=\frac{k_{4cat}[MKP3_{0}][Ep_{c}]/k_{m4}}{1+[E_{c}]/k_{m5}+[Ep_{c}]/k_{m4}+[Epp_{c}]/k_{m3}},v_{2}=\frac{k_{2cat}[MEK^{*}][Ep_{c}]/k_{m2}}{1+[E_{c}]/k_{m1}+[Ep_{c}]/k_{m2}}$$

$$v_{3}=\frac{(k_{3cat}[MKP3_{0}][Epp_{c}])/k_{m3}}{1+[E_{c}]/k_{m5}+[Ep_{c}]/k_{m4}+[Epp_{c}]/k_{m3}},v_{1}=\frac{k_{1cat}[MEK^{*}][E_{c}]/k_{m1}}{1+[E_{c}]/k_{m1}+[Ep_{c}]/k_{m2}}$$

$$v_{5}=\frac{k_{catMKPn}[MKP_{n}][Epp_{n}]}{k_{dppEn}+[Epp_{n}]}.$$

where the $\text{KDs}$ of the intermediate reactions (denoted above as $\text{k}_{\text{mi}}$) of the dual phosphorylation/dephosphorylation cycle are related to the rate constants as,

$$k_{m1}=\frac{k_{11}+kk_{2}}{kk_{1}},k_{m2}=\frac{k_{33}+kk_{4}}{kk_{3}},k_{4cat}=\frac{h_{5}}{(1+h_{5}/h_{6}+h_{33}(h_{44}+h_{5})/(h_{3}h_{4}))}$$

$$k_{m3}=\frac{h_{11}+h_{2}}{h_{1}+h_{1}h_{2}/h_{3}},k_{m4}=\frac{h_{44}+h_{5}}{h_{4}(1+h_{5}h_{6}+h_{33}(h_{44}+h_{5})/(h_{3}h_{4}))},k_{m5}\text{ }=\text{ }\frac{h_{6}}{h_{66}}.$$

**1.3. ERK and NFAT regulated Gonadotropin Subunit (GSU) transcription.** This section describes possible modes of transcriptional regulations of the $\text{GSU}$transcription, which integrate the signals delivered via $\text{NFAT}$and $\text{ERK}$as previously described (Tsaneva-Atanasova et al., 2012). Here, $\text{GSU}$is not intended to mean any specific gonadotropin α or β subunit and could be considered as any transcript for which $\text{ERK}$and $\text{NFAT}$converge at the promoter. The sequential feed-forward loop type transcriptional regulation is where an $\text{ERK}$-activated transcription factor $\text{EGR1}$activates a second transcription factor $\text{TF2}$ while both of these transcription factors increase$\text{GSU}$. In the parallel mode of regulation, both $\text{TF2}$and the dephosphorylated nuclear$\text{NFAT}_{\text{n}}$are required for initiating $\text{GSU}$transcription, but they do not interact with each other before binding to their respective promoter sites. The activation of transcription factor $\text{EGR1}$ via dually phosphorylated nuclear $\text{ERK}$and the activation of transcription factor $\text{TF2}$ via the active$\text{EGR1}$are both described using Michaelis-Menten type kinetics,

$$\frac{d[EGR1]}{dt}=\frac{k_{catTF1}[Epp_{n}]}{K_{dppETF1}+[Epp_{n}]}-d_{TF1}[EGR1],$$

$$\frac{d[TF2]}{dt}=\frac{k_{catTF2}[EGR1]}{K_{dTF1TF2}+[EGR1]}-d_{TF2}[TF2],$$

where$\text{d}_{\text{TF1}}$ and $\text{d}_{\text{TF2}}$ are the rates of de-activation or degradation of $\text{EGR1}$ and $\text{TF2}$.

Within the sequential and parallel processes, three distinct possible methods of regulations are considered (Tsaneva-Atanasova et al., 2012). An *AND* gate where both transcription factors must be bound to the promoter for transcription of the$\text{GSU}$, an *OR* gate where at least one of the two transcription factors must be bound to the promoter for transcription of the $\text{GSU}$and a co-operative (*COOP)* gate where both transcription factors must be bound to the promoter and must interact with each other after binding in order to increase transcription of the$\text{GSU}$.

The rates of $\text{GSU}$transcription can be found from the stationary-state probabilities of the transcription factor dynamics. The probability that only $\text{EGR1}$ is bound to the promoter is given by

$P_{10}=K_{TF1}[EGR1]P_{00}$,

where$\text{P}_{00}$is the probability that the promoter is unbound. Similarly, the probability that only $\text{EGR1}$is bound to the promoter is given by

$$P_{01}=K_{TF2}[TF2]P_{00}$$

And the probability that both $\text{EGR1}$and $\text{TF2}$ are bound is

$P_{11}=K_{TF1TF2}[TF2]P_{10}=K_{TF2TF1}[TF2]P_{01}$. Using $P_{00}+P_{01}+P_{10}+P_{11}=1$

so we derive the following expression

$$P_{TF1\cup TF2}=\frac{K_{TF1}[EGR1]+K_{TF2}[TF2]+K_{TF2TF1}[TF2][EGR1]}{1+K_{TF1}[EGR1]+K_{TF2}[TF2]+K_{TF2TF1}[TF2][EGR1]},$$

for the probability that at least one of the transcription factor is bound to the $\text{GSU}$promoter. Similarly, the probability that both $\text{EGR1}$ and$\text{TF2}$ are bound to the $\text{GSU}$promoter is given by

$$P_{TF1\cap TF2}=\frac{K_{TF2TF1}[TF2][EGR1]}{1+K_{TF1}[EGR1]+K_{TF2}[TF2]+K_{TF2TF1}[TF2][EGR1]}.$$

As the change in $\text{GSU}$levels $\Delta\text{[GSU]}$ between time $\text{t}$ and $\text{t+}\Delta\text{t}$ is proportional to the probability that the corresponding transcription factors are bound to the promoter, we arrive at the following ODEs for $\text{GSU}$transcription sequential cases:

$$\frac{d[GSU_{seq}^{AND}]}{dt}=\gamma\frac{[TF2][EGR1]}{1+K_{TF1}[EGR1]+K_{TF2}[TF2]+K_{TF2TF1}[TF2][EGR1]}-d_{GSU}[GSU_{seq}^{AND}],$$

$$\frac{d[GSU_{seq}^{OR}]}{dt}=\gamma\frac{K_{TF1}[EGR1]+K_{TF2}[TF2]+K_{TF2TF1}[TF2][EGR1]}{1+K_{TF1}[EGR1]+K_{TF2}[TF2]+K_{TF2TF1}[TF2][EGR1]}-d_{GSU}[GSU_{seq}^{OR}].$$

where $\text{d}_{\text{GSU}}$ is the rate of degradation for the $\text{GSU}$. On substituting $\text{TF2}$for $\text{NFAT}_{\text{n}}$ in the above expressions we obtain the analogous ODEs for the parallel cases:

$$\frac{d[GSU_{par}^{AND}]}{dt}=\frac{\gamma(([EGR1][NFAT_{n}])}{([EGR1]+K_{dTF1})([NFAT_{n}]+K_{dTF2})}-d_{GSU}[GSU_{par}^{AND}],$$

$$\frac{d[GSU_{par}^{OR}]}{dt}=\frac{\gamma([EGR1]K_{dTF1}+[NFAT_{n}]K_{dTF2}+[EGR1][NFAT_{n}]K_{dTF1}K_{dTF2})}{(1+[EGR1]K_{dTF1})(1+[NFAT_{n}]K_{dTF2})}-d_{GSU}[GSU_{par}^{OR}].$$

Finally, for the $\text{COOP}$gate, the law of mass action was used in to model the interaction between the two transcription factors (Tsaneva-Atanasova et al., 2012) while they are simultaneously bound to the promoter to obtain the following rate of $\text{GSU}$transcription for the sequential case,

$$\frac{d[GSU_{seq}^{coop}]}{dt}=K_{comp}\frac{[TF2][EGR1]}{(1+K_{TF1}[EGR1]+K_{TF2}[TF2]+K_{TF2TF1}[TF2][EGR1])^{2}}-d_{GSU}[GSU_{seq}^{coop}].$$

And the parallel case,

$$\frac{d[GSU_{par}^{coop}]}{dt}= K_{comp}\frac{[EGR1][NFAT_{n}]}{(1+K_{TF1}[EGR1]+K_{TF2}[NFAT_{n}]+K_{TF2TF1}[NFAT_{n}][EGR1])^{2}}-d_{GSU}[GSU_{par}^{coop}].$$

Finally, transcription upregulated by either $\text{NFAT}$or $\text{ERK}$pathway alone, were modelled as:

$$\frac{d[NFATdT]}{dt}=\frac{K_{comp}[NFAT_{n}]}{[NFAT_{n}]+K_{dTF1}}-d_{GSU}[NFATdT],$$

$$\frac{d[ERKdT]}{dt}=\frac{K_{comp}[TF2]}{[TF2]+K_{dTF1}}-d_{GSU}[ERKdT].$$

**1.4. Simulations with varied receptor internalization and recycling rates.**

To explore the influence of receptor trafficking on signaling we ran simulations with the LβT2 cell-trained model in which internalization and recycling rates were varied. Examples of predicted time-courses with constant and pulsatile stimulation and varied receptor internalization rates are shown in Figure 4 (main text). In a broader series we simulated responses to 10^-7^M GnRH as a constant or pulsatile stimulus (5 min period 60 min interval) setting the receptor recycling rate at 1x and varying the internalization rate from 0.03125x to 32x or fixing the internalization rate at 1x and varying the recycling rate from 0.03125x to 32x. Simulations were run for 960 min and the predicted time-courses (not shown) were used to calculate the integrated PLC response as a as a readout. This revealed, as expected, that maximal responses to sustained stimulation were greater than those to pulsatile GnRH (note the different vertical axis ranges for Supplemental Fig1A and 1B) and that with either paradigm increasing the rate of internalization reduced the response whereas increasing the rate of recycling increased it. With constant stimulation the system showed comparable sensitivity to internalization and recycling because they are equally important determinants of cell surface receptor number at equilibrium, and this is evidenced by the near symmetrical curves for internalization or recycling (open circles and filled circles in Supplemental Fig. 1A). However, with pulsatile GnRH the relationship between internalization rate and PLC activity is right shifted because agonist-induced internalization occurs only during the GnRH pulses. For the recycling rate there are opposing tendencies; recycling continues beyond the GnRH pulse and this tends to increase sensitivity to the recycling rate whereas recycling applies only to the small proportion of receptors that have internalized and this tends to reduce sensitivity to recycling rate. For the simulation parameters used here the nett effect was that pulsatile stimulation reduced sensitivity to the recycling rate (compare steepness of the filled circle plots in Supplemental Fig.1A and B).

**2. A Minimal Model for parallel signaling.**

In the following we describe a minimal cell-signaling model, where two parallel and independent pathways are activated by a common signaling molecule. Here, the effector$\text{E1}$is activated by the signal $\text{S}$ and then activates the effectors $\text{E2}$and $\text{E3}$in parallel. For simplicity, like in the LβT2 model, the pulsatile signal is represented by train of pulses of amplitude $\text{p}$*,* time period $\text{T}$ and pulse duration$\text{t}_{\text{p}}$

$$S=p(\Theta(\mathrm{mod}(t,T))-\Theta(\mathrm{mod}(t,T)-t_{p})).$$

The following coupled set of ODEs were used to model the minimal network,

$$\frac{dE1}{dt}=\frac{k_{11}S(E1_{0}-E1)}{(K_{m1}+S)}-\frac{k_{22}E1}{(K_{m2}+E1)},$$

$$\frac{dE2}{dt}=\frac{k_{1}E1(E2_{0}-E2)}{(K_{m1}+E1)}-\frac{k_{2}E2}{(K_{m2}+E2)},$$

$$\frac{dE3}{dt}=\frac{k_{1}E1(E3_{0}-E3)}{(K_{m31}+E1)}-\frac{k_{2}E2}{(K_{m32}+E2)},$$

where the deactivation rate $\text{k}_{2}$for the two parallel pathways was varied to study the differential response of the pathways to a common incoming signal $\text{S.}$

**Fitting methodology and model parameters.** The parameters of the *in silico* LβT2 model were estimated by simultaneously fitting the model predictions to a multiple data sets. The data sets included the time-course and dose-response data from $\text{ERK}$and $\text{NFAT}$translocations assays, Ca^2+^ response time-course data and the time-course data from $\text{GnRHR}$internalization assays. The parameter fitting was performed in MATLAB using the simulated annealing (*simannealbnd.m*) algorithm. The numerical integrations for both the minimal model and *in silico* LβT2 model were carried out in MATLAB using *ode45* solver. The parameter estimates for the *in silico* LβT2 model are tabulated below.

**Tables.**

**Supplementary Table 1A**

| ***LβT2 model*** |  |  |  |  |  |  |  |
| --- | --- | --- | --- | --- | --- | --- | --- |
| $k_{1}$ | 2500 $\text{min}^{\text{-1}}$ | $K_{e}$ | 0.42$\mu M$ | $k_{f3}$ | 0.013$\text{min}^{\text{-1}}$ | $h_{4}$ | 600$\mu M\text{min}^{\text{-1}}$ |
| $k_{-1}$ | 5$\mu M\text{min}^{\text{-1}}$ | $J_{in}$ | 5e4$\text{min}^{\text{-1}}$ | $k_{f4}$ | 25.62$\text{min}^{\text{-1}}$ | $h_{5}$ | 30$\text{min}^{\text{-1}}$ |
| $k_{2}$ | 0.5$\text{min}^{\text{-1}}$ | $L$ | 40$\text{min}^{\text{-1}}$ | $k_{mn}$ | 0.396$\mu M$ | $h_{6}$ | 5.16$\text{min}^{\text{-1}}$ |
| $k_{-2}$ | 1$\mu M\text{min}^{\text{-1}}$ | $P$ | 1.6e6$\text{min}^{\text{-1}}$ | $M$ | 53.5$\mu M$ | $\text{k}_{\text{catMKPn}}$ | 4.92$\mu M\text{min}^{\text{-1}}$ |
| $R_{0}$ | 0.1$\mu M$ | $A$ | 16.5$\mu M\text{min}^{\text{-1}}$ | $n$ | 3.86 | $\text{k}_{\text{dppEn}}$ | 0.1$\mu M$ |
| $k{}_{3}$ | 36$\text{min}^{\text{-1}}$ | $K_{i}$ | 7.9e-4$\mu M$ | $N_{tot}$ | 1$\mu M$ | $\text{MEK}_{t}$ | .04$\mu M$ |
| $k_{-3}$ | 30$\mu M\text{min}^{\text{-1}}$ | $K_{a}$ | 2.605$\mu M$ | $C_{cn}$ | 3 | $\text{MKP}_{\text{n}}$ | 0.09$\mu M$ |
| $\varepsilon$ | 1e-4 | $K_{d}$ | 0.38$\mu M$ | $k_{1cat}$ | 0.6$\text{min}^{\text{-1}}$ | $\text{K}_{\text{de}}$ | 0.42$\mu M$ |
| $\sigma$ | 0.8190 | $k_{d1}$ | 5.77$\mu M$ | $k_{2cat}$ | 5$\text{min}^{\text{-1}}$ | $\text{MKP}_{\text{3t}}$ | 0.18$\mu M$ |
| $V_{p}$ | 1e4$\text{min}^{\text{-1}}$ | $k_{f1}$ | 40.66$\text{min}^{\text{-1}}$ | $h_{1}$ | 2.7e3$\mu M\text{min}^{\text{-1}}$ | $\text{M}_{\text{tot}}$ | 0.9$\mu M$ |
| $K_{p}$ | 5e-6$\mu M$ | $k_{r1}$ | 0.16$\mu M\text{min}^{\text{-1}}$ | $h_{2}$ | 5.52$\text{min}^{\text{-1}}$ | $\text{k}_{\text{impE}}$ | 2.23$\text{min}^{\text{-1}}$ |
| $V_{e}$ | 1.315e5$\text{min}^{\text{-1}}$ | $k_{f2}$ | 0.0052$\text{min}^{\text{-1}}$ | $h_{3}$ | 60$\mu M\text{min}^{\text{-1}}$ | $\text{k}_{\text{impE}}$ | 0.45$\text{min}^{\text{-1}}$ |

**Supplementary Table 1B**

| ***LβT2 model*** |  |  |  |  |  |  |  |
| --- | --- | --- | --- | --- | --- | --- | --- |
| $\text{k}_{\text{catTF1}}$ | 0.03$\text{min}^{\text{-1}}$ | $\text{k}_{11}$ | 60$\text{min}^{\text{-1}}$ | $k_{3\mathrm{int}mid}$ | 0.059$\text{min}^{\text{-1}}$ | $\text{k}_{\text{e2}}$ | 20$\text{min}^{\text{-1}}$ |
| $\text{k}_{\text{catTF2}}$ | 0.045$\text{min}^{\text{-1}}$ | $\text{kk}_{2}$ | 0.6$\text{min}^{\text{-1}}$ | $\text{Km}_{1\text{intmid}}$ | 1.88$\mu M$ | $\text{k}_{-e2}$ | 5$\mu M\text{min}^{\text{-1}}$ |
| $\text{K}_{\text{dppETF1}}$ | 0.5$\mu M$ | $\text{kk}_{3}$ | 1920$\mu M\text{min}^{\text{-1}}$ | $k_{4\mathrm{int}}$ | 5.82$\mu M\text{min}^{\text{-1}}$ | $PLC_{0}$ | 0.1$\mu M$ |
| $\text{K}_{\text{dTF1TF2}}$ | 0.26$\mu M$ | $\text{k}_{33}$ | 60$\text{min}^{\text{-1}}$ | $\text{k}_{4\text{intmid}}$ | 0.24$\mu M\text{min}^{\text{-1}}$ | $l_{bas}$ | 0.17$\mu M\text{min}^{\text{-1}}$ |
| $\text{d}_{\text{TF1}}$ | 0.017$\mu M\text{min}^{\text{-1}}$ | $\text{kk}_{4}$ | 900$\text{min}^{\text{-1}}$ | $\text{Km}_{2\text{int}}$ | 50$\mu M$ | $k_{r3}$ | 4.3975$\mu M\text{min}^{\text{-1}}$ |
| $\text{d}_{\text{TF2}}$ | 0.023$\mu M\text{min}^{\text{-1}}$ | $\text{h}_{11}$ | 60$\text{min}^{\text{-1}}$ | $\text{Km}_{2\text{intmid}}$ | 50$\mu M$ |  |  |
| $\text{d}_{\text{GSU}}$ | 0.05$\mu M\text{min}^{\text{-1}}$ | $\text{h}_{33}$ | 600$\text{min}^{\text{-1}}$ | $n_{\mathrm{int}}$ | 4 |  |  |
| $\gamma$ | 0.01$\text{min}^{\text{-1}}$ | $\text{h}_{44}$ | 60$\text{min}^{\text{-1}}$ | $l_{dp}$ | 4.93 $\mu M\text{min}^{\text{-1}}$ |  |  |
| $\text{K}_{\text{comp}}$ | 0.01$\mu M$ | $\text{h}_{66}$ | 66$\mu M\text{min}^{\text{-1}}$ | $l_{\exp}$ | 2.67 $\text{min}^{\text{-1}}$ |  |  |
| $\text{K}_{\text{dTF1}}$ | 0.02$\mu M$ | $\frac{\text{fi}}{\text{Vi}}$ | 6e-5 | $l_{imp}$ | 0.01$\text{min}^{\text{-1}}$ |  |  |
| $\text{K}_{\text{dTF2}}$ | 0.2$\mu M$ | $\text{k}_{3\mathrm{int}}$ | 18.83$\text{min}^{\text{-1}}$ | $G_{0}$ | 0.5$\mu M$ |  |  |
| $\text{kk}_{1}$ | 1200$\mu M\text{min}^{\text{-1}}$ | $\text{Km}_{\text{1int}}$ | 1e-4$\mu M$ | $\text{K}_{\text{Ee}}$ | 0.1$\mu M$ |  |  |

**Supplementary Table 2**

| ***Minimal Model*** |  |  |  |  |  |
| --- | --- | --- | --- | --- | --- |
| $k_{11}$ | 1$\text{min}^{\text{-1}}$ | $K_{m31}$ | 0.1$\mu M$ | $E1_{0}$ | 0.1$\mu M$ |
| $k_{22}$ | 1$\mu M\text{min}^{\text{-1}}$ | $K_{m32}$ | 0.01$\mu M$ | $E2_{0}$ | 0.1$\mu M$ |
| $k_{1}$ | 8$\min^{-1}$ | $K_{m2}$ | 0.1$\mu M$ | $E3_{0}$ | 0.1$\mu M$ |
| $k_{2}$ | $2^{1}-2^{5}$ $\mu M\text{min}^{\text{-1}}$ | $K_{m1}$ | 1e-3$\mu M$ |  |  |

**Figure.**

**Supplemental Figure 1. Influence of varying receptor internalization and recycling rates on signaling to PLC.** Simulations were run with the LβT2 cell-trained model and with internalization and recycling rates varied (i.e. the values estimated from data and shown in Supplemental Table 1A were set at 1x and the rate constants were then varied as defined multiples of this). Responses to 10-^7^M GnRH as a constant stimulus or in pulses (5 min period 60 min interval) were modelled for 960 min. The predicted time-courses (not shown) were used to calculate the integrated PLC response as a as a system readout and the figures show the predicted PLC response integral (AUC = area under curve) for constant stimulation (Fig.1A) or pulsatile stimulation (Fig.1B) with the recycling rate at 1x and the internalization rate varied (open circles) or with the internalization rate fixed at 1x and the recycling rate varied (filled circles) as indicated on the horizontal axis. The horizontal dotted lines are the approximate maximal system outputs and the vertical arrow in Fig.1A indicates the difference between this maximum and the response obtained with internalization and recycling both at 1x. Note that this indicates a reduction of approximately 50% from the maximum for sustained stimulation whereas the corresponding reduction is only 10% for pulsatile stimulation (i.e. when comparing maximal predicted outputs to those predicted with our best estimates of the actual rates derived from wet-lab data).

References

[1] Tsaneva-Atanasova, K., Mina, P., Caunt, C.J., Armstrong, S.P. and McArdle, C.A., 2012. Decoding GnRH neurohormone pulse frequency by convergent signaling modules, J R Soc Interface. 9, 170-82.

[2] Hodgkin, A.L. and Huxley, A.F., 1952. A quantitative description of membrane current and its application to conduction and excitation in nerve, J Physiol. 117, 500-44.

[3] Li, Y.X. and Rinzel, J., 1994. Equations for InsP3 receptor-mediated [Ca2+]i oscillations derived from a detailed kinetic model: a Hodgkin-Huxley like formalism, J Theor Biol. 166, 461-73.

[4] Cooling, M.T., Hunter, P. and Crampin, E.J., 2009. Sensitivity of NFAT cycling to cytosolic calcium concentration: implications for hypertrophic signals in cardiac myocytes, Biophys J. 96, 2095-104.

[5] Markevich, N.I., Hoek, J.B. and Kholodenko, B.N., 2004. Signaling switches and bistability arising from multisite phosphorylation in protein kinase cascades, J Cell Biol. 164, 353-9.
